# Evaluating the Effectiveness of the Housing First for Youth Intervention for Youth Experiencing Homelessness in Canada: Protocol for a Multisite, Mixed Methods Randomized Controlled Trial

**DOI:** 10.2196/46690

**Published:** 2023-09-19

**Authors:** Stephen Gaetz, Ahmad Bonakdar, John Ecker, Cora MacDonald, Sophia Ilyniak, Ashley Ward, Lauren Kimura, Aranie Vijayaratnam, Emmanuel Banchani

**Affiliations:** 1 Faculty of Education, York University Toronto, ON Canada; 2 The Canadian Observatory on Homelessness, York University Toronto, ON Canada; 3 York University Toronto, ON Canada; 4 City of Toronto Toronto, ON Canada; 5 University of Toronto Toronto, ON Canada; 6 Faculty of Arts St. Francis Xavier University Antigonish, NS Canada

**Keywords:** youth homelessness, Housing First for Youth, Canada, randomized controlled trial, RCT, Making the Shift

## Abstract

**Background:**

Emerging evidence at the international level suggests that the Housing First approach could improve the housing stability of young people experiencing homelessness. However, there is a dearth of literature in Canada on whether the Housing First intervention for young people experiencing homelessness can improve outcomes including housing stability, health and well-being, and access to complementary supports. Adapted from the original Housing First model, Housing First for Youth (HF4Y) was developed in Canada as a rights-based approach tailored specifically for young people aged 16 to 24 years who are experiencing or are at risk of homelessness.

**Objective:**

The Making the Shift Youth Homelessness Social Innovation Lab is testing the effectiveness of the HF4Y intervention in Canada. The objective of this study is to determine whether the HF4Y model results in better participant-level outcomes than treatment-as-usual services for young people experiencing homelessness in 2 urban settings: Ottawa and Toronto, Ontario. Primary outcomes include housing stability, health and well-being, and complementary supports, and secondary outcomes include employment and educational attainment and social inclusion.

**Methods:**

The HF4Y study used a multisite, mixed methods, randomized controlled trial research approach for data collection and analysis. Eligible participants included young people aged 16 to 24 years who were experiencing homelessness or housing precarity. The participants were randomly assigned to either the treatment-as-usual group or the housing first intervention group. Survey and interview data in Ottawa and Toronto, Ontario are being collected at multiple time points (3-6 months) over 4 years to capture a range of outcomes. Analytic strategies for quantitative data will include mixed-effects modeling for repeated measures and logistic models. A thematic analysis will be used to analyze qualitative data based on participants’ narratives and life journeys through homelessness. Furthermore, program fidelity evaluations are conducted within each HF4Y program. These evaluations assess how well the intervention aligns with the HF4Y model and identify any areas that may require adjustments or additional support.

**Results:**

The HF4Y study has received human participant research ethics approval from the Office of Research Ethics at York University. Recruitment was conducted between February 2018 and March 2020. Data collection is expected to be completed at both sites by March 2024. A preliminary analysis of the quantitative and qualitative data collected between baseline and 24 months is underway.

**Conclusions:**

This pilot randomized controlled trial is the first to test the effectiveness of the HF4Y intervention in Canada. The findings of this study will enhance our understanding of how to effectively deliver and scale up the HF4Y intervention, with the aim of continually improving the HF4Y model to promote better outcomes for youth.

**Trial Registration:**

International Standard Randomized Controlled Trial Number (ISRCTN) ISRCTN10505930; https://www.isrctn.com/ISRCTN10505930

**International Registered Report Identifier (IRRID):**

DERR1-10.2196/46690

## Introduction

### Background

Youth homelessness has become a social problem in Canada that does not lend itself to a straightforward solution. Young people who experience homelessness (aged 13-24 years) account for approximately 20% of the total population experiencing homelessness in Canada, with 6000 to 7000 young people experiencing homelessness on any given night [1]. Pathways into youth homelessness are diverse and essentially emerge as the outcome of a complex and intricate interplay between individual and relational risk factors (eg, family crises and trauma), structural circumstances (eg, poverty and the lack of affordable housing), and systems failures (eg, fragmented service and program delivery) [2,3]. To effectively reduce and end youth homelessness, it is necessary to identify best practices and program interventions that address the unique needs of developing adolescents and young adults.

To date, studies have mostly focused on evaluating the different models of housing and support programs that serve adults experiencing homelessness, particularly those with mental health and substance use disorders [4]. Among such programs, the Pathways model known as Housing First gained currency in the 1990s, largely because of its demonstrated efficacy in reducing homelessness for individuals experiencing chronic homelessness and serious mental illness [5]. The underlying premise of the Pathways model was that individuals experiencing homelessness should be placed in housing as quickly as possible without conditions or prerequisites such as sobriety or medication adherence. The well-established, evidence-based body of research on the Pathways model lent further support to its success, with many cities adopting the model to address homelessness [6-9].

Within the Canadian context, the national At Home/Chez Soi project initiated in 2008 the largest randomized controlled trial (RCT) of the Housing First intervention, with many studies stemming from this research project [10-15]. The Mental Health Commission of Canada implemented the Housing First model in 5 cities (Vancouver, Winnipeg, Toronto, Montreal, and Moncton) with >2200 individuals who were experiencing homelessness. The 1-year and 2-year findings of the At Home/Chez Soi project across various sites provided compelling evidence that the Housing First intervention is an effective approach that helps individuals experiencing homelessness and who have serious mental illnesses exit homelessness more quickly compared with other treatment-as-usual (TAU) services [11,12,16,17].

However, as discussed by Goering et al [10], it remains unknown whether the Housing First intervention is effective and can be replicated among young adolescents with diverse racial and sociodemographic backgrounds, particularly within the Canadian context where Indigenous youth experiencing homelessness are overrepresented [3]. In addition, although Kozloff et al [14] presented promising results that the Housing First model can be “a viable intervention to promote housing stability in homeless youth with mental illness,” they suggested that modifications be made to the model to better meet the needs of youth, as their findings indicated that the application of the model to young people did not significantly improve other primary outcomes in the treatment group (eg, community integration and substance use).

With these considerations in mind, Housing First for Youth (HF4Y) was developed in Canada as an adaptation of the original Housing First model and is based on the understanding that the causes and conditions of youth homelessness are distinct from those affecting adults; therefore, the solutions must likewise be youth focused [18-22]. This means that HF4Y is designed specifically to meet the needs of young adults who are at risk of or experience homelessness by providing them with age-appropriate supports including housing. As a rights-based approach, HF4Y rests on the philosophy that “all people deserve housing and that adequate housing is a precondition for recovery” [23]. The core principles of the HF4Y model provide a framework for developing the interventions that are needed to help young people successfully transition to adulthood in a safe and planned way. These core principles are as follows [20]:

1. A right to housing with no preconditions: as a rights-based and youth-centric approach, HF4Y does not require youth to fulfill any precondition, such as sobriety or abstinence, to qualify for receiving housing support.
2. Youth choice, youth voice, and self-determination: this principle speaks to providing opportunities for youth to be in control of their own lives and encouraging them to make informed decisions when it comes to deciding where they want to live, rather than having this imposed on them (in real-world practice, eg, if a young person cannot live in cooperative housing with shared rooms, case workers need to discuss and work on a solution to accommodate the youth living on their own by finding a place that is suitable for the young person and within their budget).
3. Positive youth development and wellness orientation: as a strength-based approach, the HF4Y model extends beyond assessing risk and vulnerability by recognizing the assets and strengths of young individuals to build self-esteem and a positive sense of self.
4. Individualized, client-driven supports with no time limits: as youth have diverse pathways into homelessness, the program model is client-driven to ensure culturally appropriate services are offered to youth that also accommodate the spectrum of sexual orientation and gender identities with no time limits.
5. Social inclusion and community integration: HF4Y promotes social inclusion by building youth’s life skills and strengthening their existing relationships or by providing opportunities for them to meaningfully participate in community activities and engage with school and the labor market.

There is emerging evidence on the effectiveness of HF4Y at the international level [24-26]; however, little is known about the effectiveness of the HF4Y model in Canada, particularly considering that interventions and programs based on the HF4Y model must strictly adhere to the model’s core values and principles to qualify as an HF4Y intervention [20]. This paper describes the research protocol of a 4-year comprehensive study that will contribute to the evidence base on the effectiveness of HF4Y through a rigorous program of developmental and outcomes evaluations. This will enhance our understanding of how to effectively deliver the HF4Y intervention and what outcomes are likely to be expected, with the aim of continually improving the HF4Y model to promote better outcomes for youth.

### Making the Shift Youth Homelessness Social Innovation Lab

A key challenge in addressing youth homelessness is to determine what works, for whom it works, and in what context [27]; thus, how we frame “youth homelessness” is of paramount importance. In the Canadian context, youth homelessness refers to “the situation and experience of young people between the ages of 13 and 24 who are living independently of parents and/or caregivers, but do not have the means or ability to acquire a stable, safe, or consistent residence” [3]. The youth population experiencing homelessness is diverse, with Indigenous, 2SLGBTQA+ (2-spirit, lesbian, gay, bisexual, transgender, queer [or questioning], asexual, and additional groups), and Black youth being overrepresented, meaning that our interventions need to be implemented with a focus on equity, diversity, and inclusion [3].

Focusing specifically on the prevention of youth homelessness (ie, reducing inflow into homelessness and returns to homelessness), the Making the Shift (MtS) Youth Homelessness Social Innovation Lab is coled by A Way Home Canada and the Canadian Observatory on Homelessness, which is housed at York University. MtS supports the facilitation of sustainable exits from homelessness by identifying, developing, prototyping, testing, evaluating, and mobilizing innovations in policy and practice through the implementation of demonstration projects. Blending experimental program delivery with research and evaluation, the demonstration projects include 15 sites across Canada in 12 communities in Alberta, British Columbia, Newfoundland and Labrador, and Ontario. These projects are intended to expand the knowledge and understanding of innovative approaches to preventing and ending youth homelessness by using design thinking and producing scientific evidence to truly meet the needs of those served. The demonstration projects also help determine how and whether a proposed policy or intervention works—known as establishing “proof of concept”—which can later be used to inform policy and provide knowledge for “scaling” successful models beyond the original communities.

As part of the MtS Youth Homelessness Social Innovation Lab, 3 demonstration projects on HF4Y are currently being implemented by community agencies in Ottawa, Toronto, and Hamilton in Ontario, Canada. In each of the cities, agencies that work with youth experiencing homelessness are implementing and operating the HF4Y program, consistent with the model developed by Gaetz et al [21]. Each testing site focuses on specific populations of young people experiencing homelessness. Although Ottawa focuses on youth currently experiencing homelessness, Toronto focuses on youth exiting the child welfare system as a preventive intervention.

In Hamilton, an RCT design is not being used as it does not align with the values of Indigenous knowledge and traditions. Instead, a hybrid model is being implemented; it is called *Endaayaang* and combines the core principles of HF4Y with Indigenous ways of knowing and cultural practices. In Canada, Indigenous youth include 3 distinct cultural groups, that is, First Nations, Metis, and Inuit. Youth from these communities are overrepresented in the population of youth experiencing homelessness because of the history of colonialism (including practices aimed at eradicating Indigenous cultural traditions such as residential schools and the 1960s Scoop); involvement with child protection services; and the presence of intersecting forms of oppression, intergenerational trauma, and marginalization [28-30]. The adaptation of the HF4Y program necessitates the inclusion of Indigenous leadership in the program for it to succeed. Therefore, an Indigenous-led methodology that adopts a hybrid approach combining Western and Indigenous methodologies and ways of knowing is being used [31]. This approach combines elements from the settler-developed HF4Y model with Indigenous ways of knowing and cultural practices, rooted in the understanding that Indigenous youth must experience a sense of belonging within their community and have access to cultural and spiritual guidance from Elders and Traditional Knowledge Keepers to achieve positive outcomes.

The demonstration projects intend to help build an evidence base for youth homelessness prevention in Canada and to inform work on youth homelessness internationally. The research protocol described in this paper focuses on the models being implemented at the HF4Y sites in Toronto and Ottawa. The *Endaayaang* program model, which incorporates Indigenous epistemology, vernacular knowledge, and Indigenous-led research methodologies, will be described separately.

### Study Objectives

The HF4Y study seeks to engage relevant stakeholders and communities in a collaborative research and knowledge translation process throughout all stages of the project. The stakeholders include young people with lived experiences of homelessness, Indigenous communities, the agencies engaged in the homelessness sector and delivering interventions, and the broader network of service providers for youth experiencing homelessness in each city. On the basis of an equity lens, this study also intends to address the intersectional needs of racialized, Indigenous, and 2SLGBTQA+ youth populations as well as youth with disabilities. Together, this collaboration aims to achieve the following objectives:

1. To determine whether the HF4Y model results in better participant-level outcomes than TAU services for young people experiencing homelessness in 2 urban settings (Ottawa and Toronto) with respect to (1) housing stability; (2) health and well-being; (3) complementary supports such as social functioning and life skills (4) engagement in education, training, and employment; and (5) social inclusion.
2. To identify the critical components of the HF4Y model and what modifications are needed to effectively serve particular communities and subpopulations (eg, youth exiting the child welfare system, Indigenous youth, and 2SLGBTQA+ youth).
3. To identify the main pathways into youth homelessness or precarious housing situations and understand the key challenges young people experience in terms of recovery, the ability to thrive, and housing precarity. This will allow policies, practices, and program interventions to be improved to better serve the needs of youth experiencing homelessness.

It should be noted that the relationship between these objectives can be framed in a complementary manner, which helps improve the HF4Y program to better respond to the needs of youth experiencing homelessness.

## Methods

### Study Design

The HF4Y study uses a longitudinal, multisite, mixed methods RCT research approach for data collection and analysis. The choice of using an RCT design was consistent with the relevant body of clinical trials at the intersection of homelessness, social policy, and health [16,32,33], particularly the At Home/Chez Soi national project [10]. RCT studies are widely regarded as the highest standard for studying causal relationships between interventions and outcomes [34,35]. Despite their limitations, such as population availability, missing data, and participant dropouts [36], RCTs can successfully minimize a substantial portion of the inherent biases commonly observed in other study designs.

There is a growing recognition of the importance of integrating qualitative approaches in RCTs [37]. The choice of incorporating a qualitative inquiry in this study was intended to gain insights from youth participants during the implementation phase to better understand the effects of the HF4Y interventions and how they are experienced by recipients. Therefore, the results of the qualitative inquiry and RCT can be integrated to draw meaningful conclusions about the efficacy of the HF4Y intervention.

Each of the 2 site-specific HF4Y demonstration projects was tailored to reflect the needs of the target population as well as the unique local environments and cultural contexts at hand. At both the Ottawa and Toronto sites, 2 groups were created: an intervention group that received housing first (HF intervention) and a control group that received TAU services. At both sites, the participants randomized to the HF intervention group receive housing and support, consistent with the HF4Y program model [20,21], including the provision of a housing subsidy for the duration of the study (4 years). Youth participants are offered a range of housing options with no treatment preconditions (eg, in-place crisis, transitional, supportive, or scatter-site housing with mobile supports), and they receive an array of case management services related to housing retention, well-being, income and employment, education, and social inclusion as well as complementary supports. Participants randomized to the TAU group do not receive the HF intervention but generally have access to supports and regular housing programs in the community that are available for young people, potentially including income support, drop-ins, physical and mental health clinics, emergency shelters, and transitional housing or longer-term housing. Participants in this group are provided with an information package about both housing and supports and are invited to use the field office or participating service agency as a resource.

### Partnership and Public Involvement

The HF4Y research study actively engaged community partners and youth with a lived experience of homelessness throughout the design and implementation process. Forming a partnership with key stakeholders, including community agencies, was pivotal to advancing the goals of the study while allowing for a more meaningful collaboration between researchers and service providers. It was important that the study be delivered in partnership with local agencies, as the ultimate aim was to build practical knowledge and an evidence base for the HF4Y intervention. Combining on-the-ground knowledge with insights provided by a large body of stakeholders, including service providers, policy makers, researchers, and youth with lived experience, was an underlying ethos that undergirded the development of the original HF4Y program model guide [20].

The collaboration between researchers and 2 key organizations, namely, the Street Youth Planning Collaborative (Hamilton) and the National Learning Community on Youth Homelessness, played a central role in shaping and executing this study. In particular, the input from young individuals with lived experience was crucial and highly valued during this collaborative process. The findings of this study will also be shared with the youth participants and community partners who actively participated in the research.

### Ethical Considerations

The study’s human participant research protocol was approved by the Office of Research Ethics at York University (2017-382).

The eligible participants are provided with informed consent forms that contain information about the study, participants’ rights, expectations, potential risks and benefits, the amount of compensation, and the resources and services available for them during the study. Informed consent is also solicited from participants for each follow-up survey or interview. Participants are compensated for their participation in the research depending on the length of the sessions they attend. For the intervention group, 90-minute appointments are being remunerated with CAD $50 (US $37) per session, and shorter appointments of 30 minutes are being remunerated with CAD $25 (US $18.5) per session. For the TAU group, 90-minute appointments are being remunerated with CAD $60 (US $44.4) per session, and shorter appointments of 30 minutes are being remunerated with CAD $35 (US $25.9) per session. Qualitative interviews last approximately 60 to 90 minutes, and participants in both the intervention and control groups receive CAD $50 (US $37) per interview.

The privacy and confidentiality of participants will strictly be followed to the fullest extent possible by the law. All identifying information collected will be treated as strictly confidential. The names of the participants will not be used to identify the data at any stage of the research. Each participant will instead be identified by a number code on every survey, recording, and transcript to ensure privacy. The participants ID list, linking the participants’ names with their unique identifiers, will be kept electronically on a password-protected computer in the locked office of the principal investigator at the Canadian Observatory on Homelessness at York University. This participants ID list will be kept separate from all other data and will only be accessible to the research team enlisted in the study’s human participant research protocol. The participants ID list will be kept in the event that any participant wishes to withdraw from the study following data collection. In terms of data analysis, the research team will only be working with deidentified data, and all findings will be presented at the aggregate level. For the purpose of research dissemination, no identifying information about the participants themselves or the names of participating agencies and organizations will be shared in publications or presentations.

### Participants’ Recruitment

The recruitment process began by identifying eligible youth across the demonstration sites, which included young people aged 16 to 24 years who were experiencing homelessness or housing precarity consistent with the Canadian definition of youth homelessness [3]. However, because of concerns over participants not reaching the age of majority to be able to participate in the HF4Y research study, the age range was set between 16 and 24 years. Owing to different operational procedures, each community agency included additional criteria in terms of recruiting participants.

Potential study participants came from the pool of young people who accessed homelessness services and associated sectors (eg, child protection services, youth mental health or addictions services, law enforcement, or corrections). Each site determined additional inclusion criteria to address existing gaps in local service delivery as well as individualized recruitment strategies based on local prioritization processes and budgeting limitations. For example, the Ottawa site recruited youth aged 18 to 24 years who were experiencing homelessness, including young people living in a shelter, couch surfing, or sleeping outdoors. The Toronto site recruited youth experiencing homelessness (or at risk of experiencing homelessness) aged 17 to 24 years who had been in or were transitioning out of the provincial child welfare system.

To identify eligible participants to be recruited for the study, outreach activities began with local agencies and providers who were in contact with young people experiencing homelessness in Ottawa. Community representatives from each site developed a strategy to work with potential referral sources and inform them about the study. Strategies to ensure adequate participation in the study included seeking referrals from a wide range of services accessed by each site population. In Ottawa, these included local youth shelters, drop-in centers, city prioritization lists (ie, by-names list), and other local service agencies delivering programs for youth experiencing homelessness. In Toronto, youth were primarily referred by Youth in Transition workers who work with youth exiting the child welfare system, in addition to the types of referral sources used in Ottawa. Researchers and service delivery teams followed site-specific strategies for informing referral agencies (eg, outreach, posters, and meetings with key staff), identifying prospective participants, making contact to ascertain participant interest, and carrying out screening processes.

Recruitment for the study started in February 2018 in Ottawa and was completed in November 2019, with 86 participants enrolled in the study. In Toronto, the first participants were recruited in June 2018, and the final group of youth was admitted to the study in March 2020, with 62 youth participants in total. At the initial interview, participants were guided through an informed consent process and then asked to complete a baseline evaluation (interview and survey) before receiving a notification of the group assignment.

### Data Collection and Randomization

Survey and interview data in Ottawa and Toronto are being collected at multiple time points over the course of 4 years to capture a range of outcomes, as listed in Table 1. Data collection is expected to be completed at both sites by March 2024. The data collection procedures follow a sequential, mixed methods design [38], which involves a 2-phase process starting with the quantitative data collection, followed by a qualitative phase to help provide a deeper understanding of the survey responses and quantitative results based on participants’ personal narratives and life journeys through homelessness.

**Table 1:** Core outcome areas, domains, measures, and interval schedule.

Core outcome areas, variables, and domains			Measures and instruments		Interval
**Housing stability^a^**					
	Obtaining and maintaining housing with reduced stays in emergency shelters	Residential timeline follow-back [39]^b^		3 months^c^	
	Knowledge and skills regarding housing and independent living	Casey-Ansell Youth Life Skills Assessment (adapted) [40]		6 months	
**Health and well-being^a^**					
	Perceptions of health and well-being	World Health Organization Quality of Life-Brief Form [41]		6 months	
	Behavioral health	Global Appraisal of Individual Needs–Short Screener [42]		6 months	
	Food security	Food Security Survey [43]		6 months	
	Psychological functioning	Brief Symptom Inventory [44]		6 months	
	Substance use patterns	Ontario Student Drug Use and Health Survey [45]		6 months	
	Self-efficacy	General Self-Efficacy Scale [46]		6 months	
**Complementary supports^a^**					
	Life skills	Casey-Ansell Youth Life Skills Assessment (Adapted) [40]		6 months	
	Access to necessary nonmedical services	Diagnostic interview		6 months	
	Hope for the future	Herth Hope Index [47]		6 months	
	Legal and justice issues	Diagnostic interview		6 months	
	Resilience	The Resilience Scale-14 [48]		6 months	
**Education and employment**					
	Participation in education and training	VTLFB^d^ and education [13]		3 months	
	Educational achievement	Diagnostic interview		24 months	
	Participation in the labor force	VTLFB and employment		3 months	
	Financial security	VTLFB and income		3 months	
**Social inclusion**					
	Family and natural supports	Multidimensional Screener of Perceived Social Support		6 months	
	Sense of belonging to the community	Psychological Community Integration Scale [49]		6 months	
	Engagement in cultural and meaningful activities	Physical Community Integration Scale [50]		6 months	

^a^Primary outcomes domains.

^b^The residential timeline follow-back (RTLFB) is designed to assess various aspects of housing status and stability. In this study, it serves to incorporate point-in-time assessments and longitudinal evaluations of participants’ housing stays and transitions. It allows building a chronological record of each respondent's residential history between successive interviews. In addition, the RTLFB collects information about the type of residence, the individuals the participants are living with, and the reasons for moving in and moving out during the specified period. The records obtained from the RTLFB will be analyzed to gain insights into the specific places where young people stayed during the study, as well as the reasons for their moves in and out of different residences. This information will contribute to a comprehensive assessment of the effectiveness and impact of the HF4Y intervention on their housing stability and overall well-being.

^c^The measures and intervals listed will be used at the interval indicated to collect data over 48 months (eg, the residential timeline follow-back is being used every 3 months for 48 months).

^d^VTLFB: vocational timeline follow-back.

It was expected that baseline differences would exist across sites, reflecting the diverse demographic, socioeconomic, and ethnoracial background of participants based on regional characteristics. To achieve representativeness in the RCT sample, homogeneous sampling was used [38], which targeted several shared characteristics including age range (youth aged 16-24 years) and those who were experiencing homelessness or were at risk of homelessness. For each site, a minimum of 63 participants needed to be recruited for the study to be able to detect an effect size of 0.5 between the TAU group and the intervention group for the primary outcome domains, given α=.05 and β=.20. However, the total number of participants recruited was 148 to account for an attrition rate of 25%, following similar RCT-designed studies [11,17].

**Figure 1:**
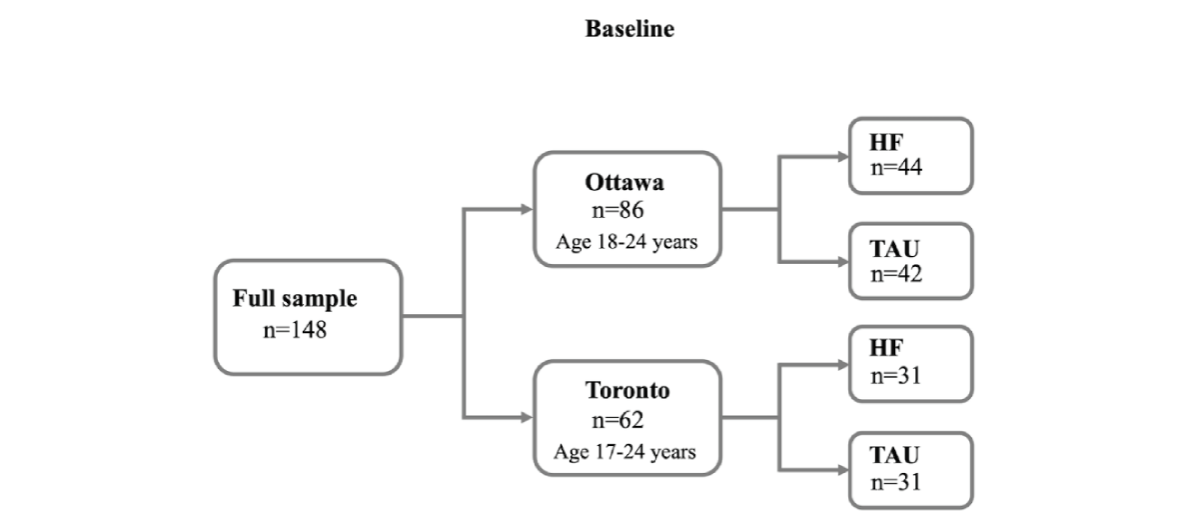
Housing First for Youth randomized controlled trial study design HF: housing first; TAU: treatment-as-usual.

The participants were assigned through strict randomization to either the TAU or HF intervention groups. Research assistants sealed assignments in envelopes ordered by participant identification number according to a randomization sequence generated through a web-based random sequence generator. Once the baseline interview was completed, the participants received a notification of the group assignment in a sealed envelope along with an information sheet. In Ottawa, 44 youths were assigned to the HF intervention group and 42 were assigned to TAU group. In Toronto, the number of participants in the HF intervention and TAU groups was equal (n=31; Figure 1). After group assignment, research assistants connected the participants assigned to the HF intervention group to the program service provider. Participants randomized to the TAU group were informed about the available services in the community. All participants were informed of the schedule of research follow-up appointments and were invited to use the research field office or the participating service agency as a resource center. All participants were provided with a pamphlet on existing services in the community.

### Housing and Supports Services

Participants randomized to the HF intervention group receive housing and support in accordance with the HF4Y model’s framework and core principles [20,21]. The program staff were extensively trained on the intervention requirements to ensure fidelity to the model. Supports offered include the provision of a housing subsidy, such that participants spend ≤30% of their income on housing based on the private rental market. Participants may also live in social, supported, or alternative housing with no treatment preconditions (eg, in-place crisis, transitional, supportive, or scatter-site housing with mobile support), in which case the rent supplement is not required. The provision of support services is not bound by any preconditions (eg, housing readiness, sobriety, and engagement in other treatment), although participants agree to be visited in their unit a minimum of once per week by program staff for a length of time that is appropriate to their level of need.

It is important to note that although a range of housing options is available in both Ottawa and Toronto, the immediate access to specific options may vary. Factors such as demand, capacity, and funding influence the availability of each option. Resource limitations or eligibility criteria may result in some options being more readily available than others.

The intervention services young people receive within the HF4Y model, including a housing rent subsidy and a range of supports delivered via housing-based case managers, are designed to bridge the financial gap and enhance young people’s ability to secure suitable housing, such as private market rentals, which they may not be able to afford on their own. In addition, housing-based case managers provide support in navigating housing processes while ensuring that young people are aware of their housing prospects. These supports include providing young people with comprehensive information about the various housing options available in the community as well as facilitating discussions with youth on the advantages and disadvantages of each option, including any potential constraints in accessing their preferred types of housing. Through these conversations, young people actively participate in the decision-making process, enabling them to select the type of housing, neighborhood, and level of services that best align with their individual needs and preferences. This means that young people can also reassess their needs and make adjustments to their housing options as required. This approach empowers young individuals, promotes informed decision-making, and supports their personal growth and development.

The HF4Y model offers a wide array of support services related to housing retention, well-being, income and employment, education, and social inclusion as well as complementary supports such as peer support that are offered by providers who are based off-site. These support services are individualized and tailored specifically to participants’ needs, preferences, and cultural backgrounds. Adapted from the Pathways model [5], the support services are provided in the home or community, using mostly intensive case management, which is appropriate for individuals with moderate mental health needs and linked them primarily to 1 case manager rather than a team. In the HF4Y program model [20-22], an intensive case management model should be established in the range of 7 to 10 youths per case manager, with 7 being the ideal number to produce the most desired, optimal outcomes for youth. Case managers work with participants to obtain and maintain housing and to navigate referrals to external services (eg, mental health support and medical services).

Compared with the HF intervention group, no active intervention is introduced to the TAU group. However, participants may continue to access existing supports and housing available in each community, including income support, drop-ins, physical and mental health clinics, emergency shelters, transitional housing, and longer-term housing. As both Ottawa and Toronto are urban centers with agencies that deliver housing services targeted to young people, it is recognized that some individuals in the TAU group may over time, through new or existing programs, access some of the same services being offered to the HF intervention group. It is also likely that the usual care service patterns will differ between cities.

### Participant Retention

Participation retention is a major concern, particularly in RCT studies where receiving maximum response rates and compliance with the research protocol is pivotal to their success. In this longitudinal study, enrolling young people experiencing homelessness and housing precarity was a particularly difficult task because of their involvement in a variety of competing activities, such as school and meeting their basic needs. In addition, barriers such as unstable housing and frequent transitions have made it difficult for youth to maintain a consistent mode of communication over time. The COVID-19 pandemic in February 2020 compounded these challenges, as it disrupted the efforts made to establish and maintain contact with youth.

Existing literature suggests strategies to minimize participant attrition, which broadly include baseline tracking procedures such as collecting detailed information on the participant’s location and follow-up procedures, including sending reminders to conduct follow-up interviews [51-53]. Consistent with the literature, this study uses an array of techniques to maintain contact with hard-to-reach participants, which include building rapport, supplementing traditional tracking methods (eg, phone, email, social media, and known associates) with new technologies, and offering financial incentives. More importantly, as data collection is still underway, a key factor is cultivating an appreciation of the contribution of the respondents as experts. Such a “participant-centered” approach [54] ensures that participants are aware of the impact of their participation in the outcomes and success of the study, thereby increasing the likelihood that they will continue to be engaged in the study. Although the existing body of research suggests varying rates of retention depending on the types of strategies used, ranging from 65% to 85% [53], this study aims to keep approximately 75% of the participants engaged in the study over 4 years.

### Outcome Areas and Measures

Building on the existing body of research including the At Home/Chez Soi national study, primary outcomes were selected to reflect the unique characteristics of youth experiencing homelessness and to measure the effectiveness of the HF4Y program interventions that are consistent with the program’s core principles and the detailed service delivery model [20,21,55]. In evaluating complex interventions, there are a range of factors that likely have an impact on housing stabilization, including sufficient and stable income, health and well-being, involvement with the justice system, involvement in education and employment, and social inclusion [56,57]. The core primary outcomes are thus selected to assess the effectiveness of HF4Y interventions on housing stability (as defined by a joint function of the number of days housed and the number of moves), health and well-being (mental and physical health status), and access to complementary supports, with secondary outcomes consisting of participation in education and employment and social functioning, as listed in Table 1 [55]. Multimedia Appendix 1 provides a comprehensive description of the measures.

In Ottawa and Toronto, interviews and surveys designed to collect quantitative data were administered at baseline, and each follow-up takes place at specific time points (3-6 months) over 4 years. At entry into the study (baseline), youth participants were interviewed using a range of questions aimed at collecting demographic data, physical health conditions, access to care, housing retention (using residential timeline follow-back), and employment (using vocational timeline follow-back; refer to Multimedia Appendix 1 for a detailed description of the interview measures). Following the interview, the participants were administered a battery of survey measures for physical and mental health and well-being, social inclusion, self-esteem and self-efficacy, resilience, hope, quality of life, and a measure reflecting complementary skills that impact the various domains (Table 1). In-person and phone-based follow-up quantitative interviews are being conducted at 3- or 6-month intervals depending on the instrument used. Since the onset of the COVID-19 pandemic, interviews and surveys with youth participants have been conducted remotely via Zoom (Zoom Video Communications) or phone. The number and timing of interview sessions were decided based on two factors: (1) the intention to evaluate longer-term outcomes and trajectories of change for each participant and (2) the recognition that participants may miss appointments owing to the nature of their situation and issues. This may be even more pronounced in the TAU group that has no interaction with the intervention, and, therefore, a potentially weaker incentive to attend appointments as scheduled.

The collection of primary data is mostly achieved through interviews conducted via Zoom or phone, with a mixture of both paper- and computer-based survey methods. Each interview appointment takes approximately 30 to 60 minutes to complete, and the participants are compensated for their time. Computer-based surveys administered through the *Qualtrics* survey platform are entered into a secure central database using wireless technology. Hard-copy data are stored securely following double-lock procedures at each site, and electronic backups are stored on cloud-based, secure, institutional network–based storage. Several strategies are being used to ensure optimal data quality: (1) using validated and reliable tools for susceptible youth populations; (2) using a cross-site protocol for data management, data entry, quality checking, and cleaning; (3) practicing common data-checking routines at each site through monthly multisite data quality committee meetings; (4) training interviewers for face-to-face and web conferencing interaction with participants; and (5) fielding interviewer questions centrally and making decisions where necessary across both RCT sites.

Following a sequential mixed methods approach [38], a subset of 20 youths per group at each RCT site was randomly selected (10 participants from the HF intervention group and 10 from the TAU group) to participate in a narrative interview, once at 6 months and later at 18 months. Demographic data of participants successfully recruited into the qualitative study were then assessed to ensure balance in demographic variables including age, gender identity, and ethnoracial background.

The purpose of this qualitative research inquiry is (1) to supplement the quantitative data collected with respect to the effectiveness of the HF4Y program and (2) to help identify areas that need improvement for future scaling of the HF4Y program. This qualitative approach is also intended to allow us to understand the lived and living experiences of study participants in their own words through life story (or “narrative”) interviews. For the large majority of youth participants, homelessness is not the product of a single event, but it is seen as a journey in which individual agency and life decisions take center stage. The qualitative interviews follow a semistructured format and include questions that are mapped onto the quantitative outcome domains. The follow-up interviews help understand how youths’ lives have changed during the RCT and what things may have contributed to those changes. These interviews specifically inquire about the types of services within and outside the HF4Y program that participants are currently receiving. They also explore the types of services participants require but are not currently receiving as well as their perspectives on the helpfulness or limitations of the services they are accessing.

### Analytical Procedures

HF intervention and TAU groups will be compared within each RCT site; although if it is determined that no significant differences exist between the 2 sites in terms of sociodemographic characteristics and outcome measures collected at baseline, the TAU and intervention groups will be combined accordingly across sites to increase the power of the study. There will be an interim analysis using 2-year quantitative data, with the final analyses based on the 4-year data. It is expected that certain participants might not adhere to the protocol or could discontinue their involvement owing to health issues or fatigue arising from repeated assessments. As deviations from the protocol are inevitable and likely to occur in real-time practice [58-60], there exist a number of approaches that can preserve the integrity of the randomization process including an intention-to-treat analysis, which considers all participants initially randomized in the study.

In addition, certain participants may be more likely not to respond to surveys, or certain questions might be more likely to have missing responses. With respect to missing data, 3 common mechanisms can be observed: missing completely at random, missing at random, and missing not at random [61,62]. For data missing completely at random, single imputation methods can be used to address the missing values, albeit with a tendency to provide conservative estimates of the treatment effect; for example, missing values can be replaced with the last observed values or the mean values. For data missing at random and missing not at random, multiple imputation can be used, which produces a more accurate and less biased estimate of the treatment effect [63].

For continuous outcome measures, differences between the intervention and control groups will be examined by using mixed-effects modeling for repeated measures. For binary outcomes, logistic models will be used, and negative binomial modeling will be used for all count outcome measures to account for any possible overdispersion in the data. Sociodemographic characteristics collected at baseline will be entered into the models as covariates, and the interaction between time and intervention will be considered to capture the interaction effects. All quantitative analyses will be conducted in SPSS (IBM Corp) and R studio (Posit) software packages with power set at 80% (α=.05 and β=.20) to detect an effect size of 0.5 between the TAU and intervention groups for the primary outcome domains.

Qualitative data will be assessed through thematic analyses to identify common themes related to outcomes and to analyze the underlying intervention process, consistent with the approaches suggested by Miles et al [64] and Saldaña [65]. The qualitative data provide a rich source of the narratives regarding young people’s experiences with homelessness, housing instability, and interaction with the HF4Y program itself. Interviews will be transcribed and imported into NVivo (QSR International) software for analysis. Rather than focusing on preexisting theoretical constructs using an a priori method, an emergent, bottom-up approach will be used for coding [64]. The process of coding will shift back and forth from the key narratives provided by the participants to the researcher’s interpretation of the meaning of those narratives, which will be documented through the researcher’s analytic memos. Recurring patterns gleaned from the codes will be grouped as themes and will be thematically classified under broader themes. The final set of themes will be summarized and presented in a table, where representative quotes from transcripts will be paired with themes as textual evidence.

In addition to the quantitative and qualitative analyses, each agency is responsible for completing monthly reports that provide important metrics for all participating youth, including demographic information and key indicators such as the number of youth housed during the month and the number of youth who exit a program. Furthermore, program fidelity evaluations are conducted within each HF4Y program. These evaluations assess how well the intervention aligns with the HF4Y model and identify any areas that may require adjustments or additional support.

### Data Access

Quantitative data are entered directly into laptops configured specifically for the project and are stored using a contracted service provider who will manage data storage for the study on an off-site, centralized server with high levels of physical network security. No data are stored on the hard drive, and after entry, the hard copies are kept in secure storage at each site. Access to the data is limited to authorized users only, using a multilevel permission protocol.

## Results

The HF4Y study has been registered with the International Standard Randomized Controlled Trial Number (ISRCTN10505930). Human participant research ethics approval has been obtained from the Office of Research Ethics at York University. Recruitment was conducted between February 2018 and March 2020. Data collection is expected to be completed at both sites by March 2024, and preliminary analysis of data collected between baseline and 24 months is underway. The preliminary findings of the study will be published through public-facing reports at 24 months, and these results will be generated from deidentified and aggregate-level data (final results will be reported at 48 months). These reports will be made publicly available for the broader network of community partners and all levels of government (federal, provincial, and municipal), and they will be hosted virtually for a larger readership. Furthermore, several peer-reviewed, scholarly publications will be published based on the study’s findings in top-tier journal outlets with an open-access option. During the course of the study, research vignettes and blog posts will highlight the ongoing progress of the research project and discuss the lessons learned, and this will be followed by a number of webinars and presentations at major national and international conferences. In addition, as part of the MtS project and in the context of what we have learned from implementation science [66-70], a robust training and technical assistance capacity has been developed to enable the spread and scaling of the HF4Y program. In particular, an iterative interaction between research and practice enhances service design and contributes to the continuous quality improvement of training and technical assistance.

## Discussion

### Strengths and Limitations

As the first pilot RCT to test the effectiveness of the HF4Y intervention in Canada, this study’s strength lies in its approach to engaging participants and community partners in the study design while using a sequential, mixed methods design during the quantitative phase, followed by a qualitative phase. However, this study has some limitations. A larger sample size would make the study’s findings more robust in terms of aggregate-level analyses. The current sample size could potentially limit the generalizability of our results. Participant dropouts, missed intervals, and noncompliance between the HF intervention and TAU groups could also affect the outcomes. Furthermore, RCT studies have limitations including issues related to low sample sizes, external validity, and missing data, which are applicable to this study.

### Public Health Implications

The findings of this study will help build an evidence base on the impact of HF4Y through a rigorous program of developmental and outcomes evaluations. The findings will have potential public health implications for youth experiencing homelessness or housing precarity, as housing stability is central to preventing homelessness and addressing the range of physical and mental health issues resulting from housing instability [55]. Consistent with the emerging evidence at the international level [24,26], we anticipate an increase in housing stability and a reduction in homelessness, followed by longer periods of housing retention in the HF4Y intervention groups compared with the TAU group. This could lead to enhanced physical and mental well-being, improved social functioning, and increased community integration while allowing participants to meaningfully engage in education and employment opportunities. In addition, improved housing quality and increased housing stability are likely to lead to a reduction in the use of hospitals and emergency services as well as improved clinical outcomes, such as psychiatric symptoms and substance use, and reduced involvement in the criminal justice system.

### Conclusions

Although there is emerging evidence on the efficacy of HF4Y globally [24-26], there is limited knowledge about its effectiveness specifically in Canada. This knowledge gap is significant because the HF4Y interventions and programs in Canada must adhere closely to the model’s core values and principles to be recognized as true HF4Y interventions. This study will contribute to our understanding of how to effectively deliver and scale up the HF4Y intervention, with the aim of continually improving the HF4Y model and promoting better outcomes for youth.

The HF4Y pilot study will provide an evidence base for transforming policy and program interventions to address youth homelessness. This requires a shift away from solely managing the crisis and toward prioritizing prevention, which entails supporting those who are experiencing or at risk of homelessness to regain stable housing. By empowering individuals to sustain their housing, the HF4Y demonstration project actively seeks to engage youth in education and cultivate their sense of inclusion in the community, thereby creating a foundation for long-term health and well-being.

## Appendix

Multimedia Appendix 1 Psychometric descriptions of the measures used in the randomized controlled trial study.

## Abbreviations

2SLGBTQA+: 2-spirit, lesbian, gay, bisexual, transgender, queer (or questioning), asexual, and additional groups
HF: housing first
HF4Y: Housing First for Youth
MtS: Making the Shift
RCT: randomized controlled trial
TAU: treatment-as-usual

## Data Availability

The data sets generated and analyzed during this study are available upon request. External researchers must make a formal request to use the data sets and agree to the policies and procedures set forth by the Making the Shift. Inquiries and requests can be sent to the corresponding author.
