# Supplementary material for: Evaluating the Effectiveness of the Housing First for Youth Intervention for Youth Experiencing Homelessness in Canada: Protocol for a Multisite, Mixed Methods Randomized Controlled Trial
Source: JMIR Res Protoc. 2023 Sep 19;12:e46690. doi: 10.2196/46690 (PMC10548326; doi:10.2196/46690)
Supplement: Multimedia Appendix 1 [file resprot_v12i1e46690_app1.docx]

**Appendix A: Psychometric descriptions of measures used in the RCT study**

| **Measure** | **Description** |
| --- | --- |
| **Demographics, Service, and Housing History Form**  **(DSHH)** | The DSHH questionnaire was developed for use in the At Home/Chez Soi study using demographic items from the 2006 Canada Census, the Toronto Board of Education 2006 Student Census, and the Community Mental Health Evaluation Initiative (CMHEI). The housing vocational and service use history items were developed in consultation with content experts from the National Research Team and the Cross-site Measurement Task Group. |
| **Physical Health Conditions** | A series of questions about the physical health status of participants based on the At Home/Chez Soi study. |
| **Access to Care** | Homeless individuals have elevated rates of comorbid physical health conditions compared to  the general population and yet have inadequate healthcare due to problems with access. In the study we will be measuring access issues and reasons for problems with access using specific questions based on the At Home/Chez Soi study. |
| **Residential Timeline Follow-Back (RTLFB)** | Designed to assess all dimensions of housing status and stability, the RTLFB incorporates both point-in-time assessments and longitudinal evaluations of housing and transitions to build a chronological record of each respondent’s residential history for the period between successive interviews. |
| **Vocational Timeline Follow-Back**  **(VTLFB)** | Similar to the RTLFB but related to vocational history. |
| **Interviewer Impressions Items**  **(III)** | The purpose of these items is to note observations of circumstances at the time of the interview which could indicate validity problems with the information collected. |
| **Survey Measures** |  |
| **World Health Organization Quality of Life—Brief Form (WHOQoL-BREF)** | The aim of the WHOQOL**—**BREF was to develop an international cross-culturally comparable quality of life assessment instrument. It assesses the individual's perceptions in the context of their culture, and value systems, and their personal goals, standards and concerns. |
| **Youth Life Skills Assessment**  **(YLSA)** | Based on the Ansel-Casey Life Skills Assessment, this abbreviated youth-centred tool assesses life skills youth need for their well-being, confidence, and safety as they navigate high school, postsecondary education, employment, and other life milestones. The assessment is made up of several items categorized within three areas: skills, knowledge, and awareness. |
| **Global Appraisal of Individual Needs – Short Screener**  **(GAIN-SS)** | The GAIN-SS serves as a short screen for general populations to quickly and accurately identify clients who have one or more behavioural health disorders (e.g., internalizing or externalizing psychiatric disorders, substance use disorders, and crime or violence problems), and would benefit from further assessment or referral for these issues. It also rules out those who are not be identified as having behavioural health disorders. |
| **Food Security Survey**  **(FSS)** | The FSS focuses on self-reports of uncertain, insufficient, or inadequate food access due to limited financial resources, and the compromised eating patterns and food consumption that may result. |
| **Ontario Student Drug Use and Health Survey—Form A, Grades 9-12 (Substance Use Patterns)** | Topics in the OSDUHS Form A (substance use patterns) include tobacco, alcohol, and other drug use, harmful consequences of use, mental health indicators, physical health indicators, health care utilization, body image, gambling and video gaming problems, violence and bullying, school safety, school climate, criminal behaviours, and socio-demographics. |
| **Brief Symptom Inventory**  **(BSI)** | The BSI or the Brief Symptom Inventory is an instrument that evaluates psychological distress and psychiatric disorders in people. The BSI collects data reported by patients for evaluation. The test can be used for areas such as patient progress, treatment measurements, and psychological assessment. Externalizing and internalizing symptoms are measured as well as an overall score of functioning. |
| **General Self-efficacy Scale**  **(GSES)** | The General Self-Efficacy Scale is a short psychometric scale that is designed to assess optimistic self-beliefs related to coping with a variety of difficult demands in life. In contrast to other scales that were designed to assess optimism, this one explicitly refers to personal agency, meaning the belief that one’s actions are responsible for successful outcomes. Perceived self-efficacy is a prospective and operative construct. |
| **Herth Hope Index**  **(HHI)** | The HHI aims to measure two spheres of hope (generalized and particularized) and six dimensions: contextual, affective, cognitive, behavioural, affiliative, and temporal. In Herth's version, these six dimensions are combined into three subscales: “inner sense of temporality and future,” “inner positive readiness and expectancy,” and “interconnectedness with self and others.” |
| **Resilience Scale-14**  **(RS-14)** | The RS-14 was developed to evaluate the levels of resilience (the ability to bounce back or recover from stress) in the general population. The 14-item scale was developed by retaining the most reliable items from the 25-Item Resilience Scale. |
| **Multidimensional Screener of Perceived Social Support**  **(MDSPSS)** | The MDSPSS is a brief tool designed to measure perceptions of support from three sources of interpersonal relationships: family, friends, and a significant other. |
| **Community Integration Scale (CIS)** | This survey measures the degree to which a person is participating in and feels a sense of belonging to their community. There are two subscales: the Physical Integration Scale (physical presence in one’s community) and the Psychological Integration Scale (sense of belonging in the community). |
